# PD-L1 and FOXP3 expression in high-grade squamous intraepithelial lesions of the anogenital region

**DOI:** 10.18632/oncotarget.28715

**Published:** 2025-04-24

**Authors:** Humberto Carvalho Carneiro, Rodrigo de Andrade Natal, José Vassallo, Fernando Augusto Soares

**Affiliations:** ^1^Instituto D’Or de Pesquisa e Ensino (IDOR), São Paulo 04501-000, Brazil; ^2^Department of Anatomic Pathology, Rede D’Or, São Paulo 04321-120, Brazil

**Keywords:** HPV, high-grade intraepithelial lesion, immune evasion, PD-L1, FOXP3

## Abstract

Host immunosurveillance is an important factor in the progression of high-grade squamous intraepithelial lesions (HSIL) into high-risk human papillomavirus (HR-HPV)-related squamous cell carcinoma. Immune escape by forkhead box protein P3 (FOXP3+) immunoregulatory T cells and the programmed death-ligand 1 (PD1/PD-L1) axis, mechanisms best described in the context of invasive neoplasms, may play a role in the evolution of pre-malignant lesions. This morphological study aimed to characterize the inflammatory response and expression of FOXP3 and PD-L1 in anal, vulvar, and penile HSILs and compare them with those in low-grade SILs co-infected with HR-HPV (LSIL_HR_). The study group comprised 157 samples from 95 male and 55 female patients (median age = 35.5 years), including 122 HSILs and 35 LSILs_HR_. Dense inflammatory infiltrates and high counts of FOXP3^+^ cells were significantly more frequent in patients with HSILs than in those with LSILs_HR_ (*p* = 0.04 and 0.02, respectively). HSILs also exhibited higher PD-L1 expression (*p*_adj_ < 0.01 and < 0.01 for the SP142 and 22C3 clones, respectively), based on the Poisson generalized linear model. In addition, concordant higher PD-L1 expression was observed in cases with a greater number of FOXP3+ cells (*p* < 0.05). Our findings indicate a putative role of transcriptionally active HR-HPV in evoking an inflammatory response and immune evasion in the early phases of carcinogenesis in a subset of non-cervical anogenital HSILs.

## INTRODUCTION

High-risk human papillomavirus (HR-HPV) is the etiological factor of 90%, 70%, and 60% of squamous cell carcinomas (SCCs) of the anal canal, vulva, and penis, respectively [1]. HR-HPV-associated SCC (SCC_HPV+_) evolves from a pre-invasive state, namely, a high-grade squamous intraepithelial lesion (HSIL), which is morphologically indistinguishable regardless of the site and sex [2].

Immunosurveillance, defined as the ability of the immune system to detect and eliminate non-self-antigens, including viral or neoantigens expressed by pre-cancer or cancer cells, is paramount in the progression of epithelial cells infected with HR-HPV to HSIL and then to SCC_HPV+_. This process usually takes years or decades to occur. In the case of HPV-mediated neoplasms, the mucosa, skin, and the innate immune system, particularly the T-cell-mediated immune response, are crucial elements in immune control, preventing the persistence of the infection, which is necessary for tumor progression [3–8].

Furthermore, HSILs spontaneously regress within 6–12 months in 30–50% of cases [9–11]. The risk of progression to SCC_HPV+_ varies across different series and is usually low for immunocompetent individuals: approximately 11%, up to 9.7%, and 2–30% of anal, vulvar, and penile HSILs, respectively, can progress to SCC_HPV+_ [9, 12–14]. However, individuals with immune deficiency/dysregulation (for example, people infected with human immunodeficiency virus (HIV), solid organ transplant recipients, and patients with autoimmune diseases), have a significantly greater risk of developing anogenital SCC_HPV+_ [15–19].

Immune inhibitory mechanisms evoked by HR-HPV can occur during the host response to infection and are essential for tumor development. In the early phases of infection, HPV oncoproteins E6 and E7 can induce Langerhans cells anergy and displacement in the epithelium, along with dysfunctional activity of natural killer (NK) cells and inhibition of the T_h1_-mediated response of the adaptive immune system, which is essential for the immunological clearance of HR-HPV by cytotoxic CD8^+^ T cells [5, 11, 20]. In addition, the accumulation of forkhead box protein P3 (FOXP3) immunoregulatory T cells (FOXP3^+^T_regs_) can suppress T-cell and NK cell functions. An increased number of FOXP3^+^T_regs_ is associated with persistent HR-HPV infection and progression to SCC_HPV+_ in cervical models [5, 11, 20].

Programmed cell death protein 1 (PD1) and its ligand, programmed cell death-ligand 1 (PD-L1), are the chief immune checkpoint molecules implicated in the downregulation of T-cell-mediated inflammatory responses in physiological and pathological conditions [21, 22]. In a malignant tumor microenvironment, PD1 on activated T cells binds to PD-L1, expressed either by tumor cells or macrophages, dendritic cells, and other lymphocytes, to suppress T-cell receptor signal transduction, decreasing T-cell activity and promoting immune evasion by cancer cells [21, 23–25]. The PD1/PD-L1 axis has been almost exclusively described in the context of advanced-stage cancers due to its predictive value. Scarce studies on non-invasive neoplasms suggest that PD1/PD-L1 can promote tumor progression [26–31]. In cervical HSILs, PD-L1 is frequently expressed in dysplastic epithelial cells and inflammatory cells [32] and has been associated with progression to SCC and metastasis [31].

This morphological study aimed to characterize inflammatory responses and PD-L1 and FOXP3 expression in non-cervical anogenital HSILs and compared them with those in low-grade SILs co-infected with HR-HPV (LSIL_HR_), correlating with the main clinicopathological features.

## RESULTS

### Clinicopathological findings

Of the 181 cases that fulfilled the inclusion criteria, paraffin blocks with sufficient tissue for sectioning were available in 157 cases. The study group comprised 95 male (63.3%) and 55 female (36.7%) participants, with a median age of 35.5 years (range 25.0–50.0 years). In terms of topography, anal lesions were more common (*n* = 90, 57.3%), followed by penile (*n* = 37, 23.6%) and vulvar (*n* = 30, 19.1%) lesions. This study included 122 cases of HSILs (77.7%) and 35 cases of LSILs_HR_ (22.3%). The median lesion size was 6.0 mm (range 4.0–13.0 mm). The most frequent lesion pattern was condylomatous (*n* = 62, 39.5%), followed by verrucous (*n* = 52, 33.1%) and flat (*n* = 43, 27.4%). Multifocal lesions were present in 51 cases (32.5%) and unifocal lesions in 106 cases (67.5%). Recurrence was observed in 7 (4.7%) patients. The main clinicopathological features are summarized in Table 1.

**Table 1:** Clinicopathological features distributed by topography

	All patients	Topography			*p*-value
		Anus	Penile	Vulva	
Gender					
Male (%)	95 (63.3)	59 (39.3)	36 (24.0)	0 (0.0)	<0.01
Female (%)	55 (36.7)	27 (18.0)	0 (0.0)	28 (18.7)	
Age (range)	35.5 (25.0–50.0)	35.5 (25.0–50.0)	28.0 (25.0–39.0)	45.5 (34.8–59.3)	<0.01
	All cases				
Diagnosis					
HSIL (%)	122 (77.6)	69 (43.9)	26 (16.5)	27 (17.2)	0.15
LSIL_HR_ (%)	35 (22.4)	21 (13.4)	11 (7.0)	3 (2.0)	
Size (mm)	6.0 (4.0–13.0)	8.0 (4.0–14.0)	4.0 (3.0–6.0)	8.0 (6.0–16.5)	<0.01
Histological pattern					
Condilomatous (%)	62 (39.5)	48 (30.5)	11 (7.0)	3 (2.0)	<0.01
Flat lesion (%)	43 (27.3)	29 (18.4)	5 (3.2)	9 (5.7)	
Verrucous (%)	52 (33.2)	13 (8.3)	21 (13.4)	18 (11.5)	
Focality					
Unifocal (%)	106 (67.4)	55 (35.0)	31 (19.7)	20 (12.7)	0.05
Multifocal (%)	51 (32.6)	35 (22.3)	6 (3.9)	10 (6.4)	
Recurrence	7 (4.7)	4 (2.7)	1 (0.7)	2 (1.3)	–

Vulvar lesions tended to present in patients at older age than at other age groups (45.5 (34.8–59.3) vs. 28.0 (25.0–39.0) for penile lesions and 35.5 (25.0–50.0) for anal lesions, *p*_adj_ < 0.01 and *p*_adj_ = 0.07, respectively). Penile lesions had the smallest median size, while anal and vulvar lesions were similar in size (4.0 (3.0–6.0) vs. 8.0 (6.0–16.5) and 8.0 (4.0–14.0), respectively, *p*_adj_ < 0.01 for both). Anal lesions showed predominantly condylomatous and flat architectural patterns, whereas penile and vulvar lesions presented mostly with verrucous architecture (*p* < 0.01). No correlation was found between topography and focality (*p* = 0.05).

### P16 and HPV *in situ* hybridization

P16 block-type positivity was observed exclusively in HSILs (*n* = 120, 76.4%), while all LSILs_HR_ cases showed no stain or only focal expression (*p* < 0.01). HPV *in situ* hybridization (ISH) was positive in 67.6% of HSILs, and no significant association was found between ISH positivity and p16 expression (*p* = 0.07).

### Analysis of the inflammatory infiltrate

Overall, type 1 inflammatory infiltrate was the most frequent (47.1%), followed by types 2 (31.2%) and 0 (21.7%). HSILs presented a higher inflammatory response than LSILs_HR_ (*p* = 0.04) (Table 2). Brisk, type 2 inflammatory infiltrate was more often found in older patients, larger lesions, and the vulva (*p* = 0.02, *p* = 0.04, and *p* < 0.01, respectively). Poisson generalized linear model (GLM) yielded an independent association between HSILs and type 2 inflammatory infiltrate (*p*_adj_ = 0.04), with no interference from patient age, lesion size, or topography, indicating that high grade was the only feature that correlated with the greater intensity of the inflammatory infiltrate. Furthermore, no significant association was found between inflammation and focality or recurrence (*p* = 0.20).

**Table 2:** P16, ISH, and inflammatory infiltrate features according to diagnosis

	All cases	Diagnosis		*p*-value
		HSIL	LSIL_HR_	
P16				
Block (%)	120 (76.4)	120 (76.4)	0 (0.0)	<0.01
Focal (%)	29 (18.5)	1 (0.6)	28 (17.9)	
Negative (%)	8 (5.1)	1 (0.6)	7 (4.5)	
HPV ISH				
Positive (%)	141 (89.9)	106 (67.6)	35 (22.3)	0.02
Negative (%)	16 (10.1)	16 (10.1)	0 (0.0)	
Inflammatory type				
Type 2 (%)	49 (31.2)	41 (26.1)	8 (5.1)	0.04
Type 1 (%)	74 (47.1)	60 (38.2)	14 (8.9)	
Type 0 (%)	34 (21.7)	21 (13.4)	13 (8.3)	
T/B ratio				
CD3 >CD20 (%)	132 (84.1)	100 (63.7)	32 (20.4)	0.50
CD3 = CD20 (%)	12 (7.6)	11 (7.0)	1 (0.6)	
CD3 <CD20 (%)	13 (8.3)	11 (7.0)	2 (1.3)	
CD4/CD8 ratio				
CD4 >CD8 (%)	127 (80.9)	104 (66.2)	23 (14.7)	0.02
CD4 = CD8 (%)	20 (12.7)	11 (7.0)	9 (5.7)	
CD4 <CD8 (%)	10 (6.4)	7 (4.5)	3 (1.9)	
FOXP3 (cells/HPF)	15.0 (3.0–35.0)	16.0 (4.0–38.0)	8.0 (1.5–21.0)	0.02

Regarding infiltrate composition, we found a T-cell predominant inflammatory response, with CD3^+^ cell counts surpassing CD20^+^ cell counts in most penile and vulvar cases. In contrast, the CD20 count was often equal to or higher than CD3 count (*p* < 0.01) in anal lesions. The majority of HSILs had a CD4 >CD8 proportion compared to LSILs_HR_, which in turn had more cases with CD4 ≤CD8 (*p* = 0.02). CD4 >CD8 was associated with p16 block positivity (*p* = 0.01) but not with ISH (*p* = 0.56).

### FOXP3 and PD-L1

The median count of FOXP3^+^ cells on a hotspot was 15.0 (3.0–35.0 cells/high power field (HPF)) and was higher in HSILs than in LSILs_HR_ (15.5 (4.5–38.5) vs. 8.5 (1.5–21.0), *p* = 0.02), as well as in vulvar lesions (*p*_adj_ < 0.01) (Table 3). The FOXP3 count also increased as the inflammatory score moved from type 0 to type 1 and then to type 2 inflammatory infiltrate (*p* < 0.01) and correlated with CD4 >CD8 (*p* = 0.02). By applying the GLM, the FOXP3^+^ cell count was independently associated with all variables mentioned above (*p*_adj_ < 0.01).

**Table 3:** FOXP3 and PD-L1^*^ expression correlation with main clinicopathological features

	FOXP3 cells (cells/HPF)	*p*-value	PD-L1 (SP142) (%/area)	*p*-value	PD-L1 (22C3) (%/area)	*p*-value
Topography						
Anus	12.0 (3.3–25.0)	0.02	0.0 (0.0–5.0)	0.40	1.0 (0.0–10.0)	0.40
Penis	16.0 (2.8–32.5)		0.0 (0.0–1.0)		3.0 (0.0–10.0)	
Vulva	39.5 (5.5–61.8)		0.0 (0.0–4.3)		10.0 (0.0–30.0)	
Diagnosis						
HSIL	16.0 (4.0–38.0)	0.02	0.0 (0.0–5.0)	0.02	3.0 (0.0–20.0)	<0.01
LSIL_HR_	8.0 (1.5–21.0)		0.0 (0.0–0.0)		0.0 (0.0–4.0)	
Inflammatory type						
Type 2	38.0 (26.0–61.0)	<0.01	5.0 (0.0–10.0)	<0.01	20.0 (10.0–30.0)	<0.01
Type 1	13.0 (4.0–25.0)		0.0 (0.0–2.0)		1.5 (0.0–8.8)	
Type 0	1.0 (0.0–4.8)		0.0 (0.0–0.0)		0.0 (0.0–0.0)	
T/B ratio						
CD3 >CD20 (%)	13.0 (3.0–35.0)	0.62	0.0 (0.0–5.0)	0.98	3.0 (0.0–10.0)	0.30
CD3 = CD20 (%)	22.5 (6.5–48.3)		0.0 (0.0–6.3)		0.0 (0.0–3.3)	
CD3 <CD20 (%)	15.0 (5.0–28.0)		0.0 (0.0–1.0)		2.0 (0.0–10.0)	
CD4/CD8 ratio						
CD4 >CD8 (%)	16.0 (5.0–38.0)	0.02	0.0 (0.0–4.0)	0.95	2.0 (0.0–10.0)	0.45
CD4 = CD8 (%)	4.5 (0–26.3)		0.0 (0.0–5.0)		3.0 (0.0–10.0)	
CD4 <CD8 (%)	4.0 (2.0–9.3)		0.0 (0.0–7.5)		0.0 (0.0–4.5)	
FOXP3 (cells/HPF)	–	–	r^2^ = 0.39	<0.01	r^2^ = 0.57	<0.01
PD-L1 (SP142) (%/area)	–	–	–	–	r^2^ = 0.65	<0.01

^*^PD-L1 expressed as continuous score.

**Figure 1:**
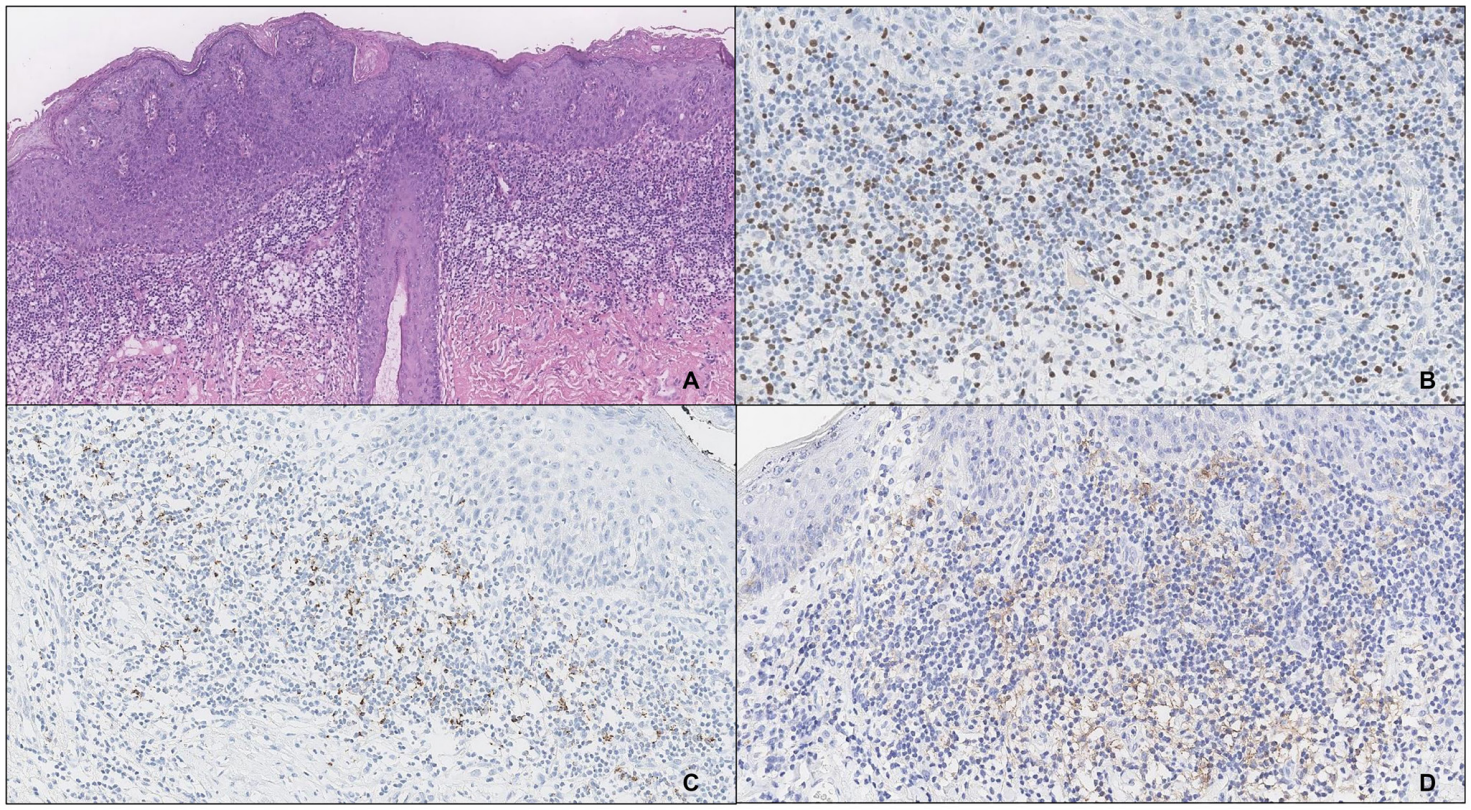
An example of vulvar HSIL with type 2 inflammatory infiltrate (**A**), high counts of FOXP3^+^ cells (40x) (**B**) and expression of PD-L1, SP142 (**C**) and 22C3 (**D**) in the IC.

**Figure 2:**
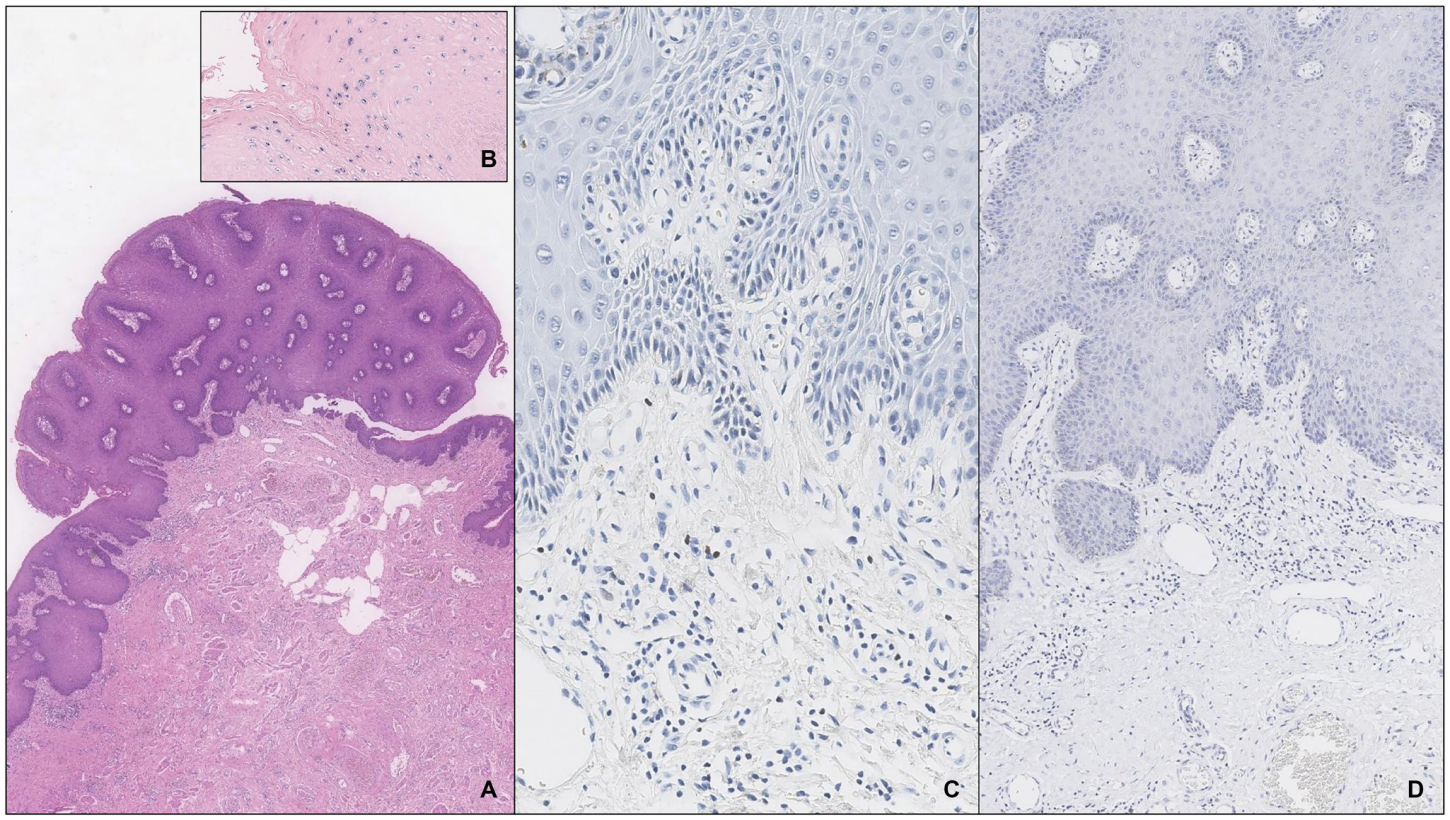
A penile condilomatous LSIL with type 0 infiltrate (**A**) and coinfection with HR-HPV by ISH (**B**). There are rare FOXP3^+^ cells (**C**) and no expression of PD-L1_22C3_ (**D**).

**Figure 3:**
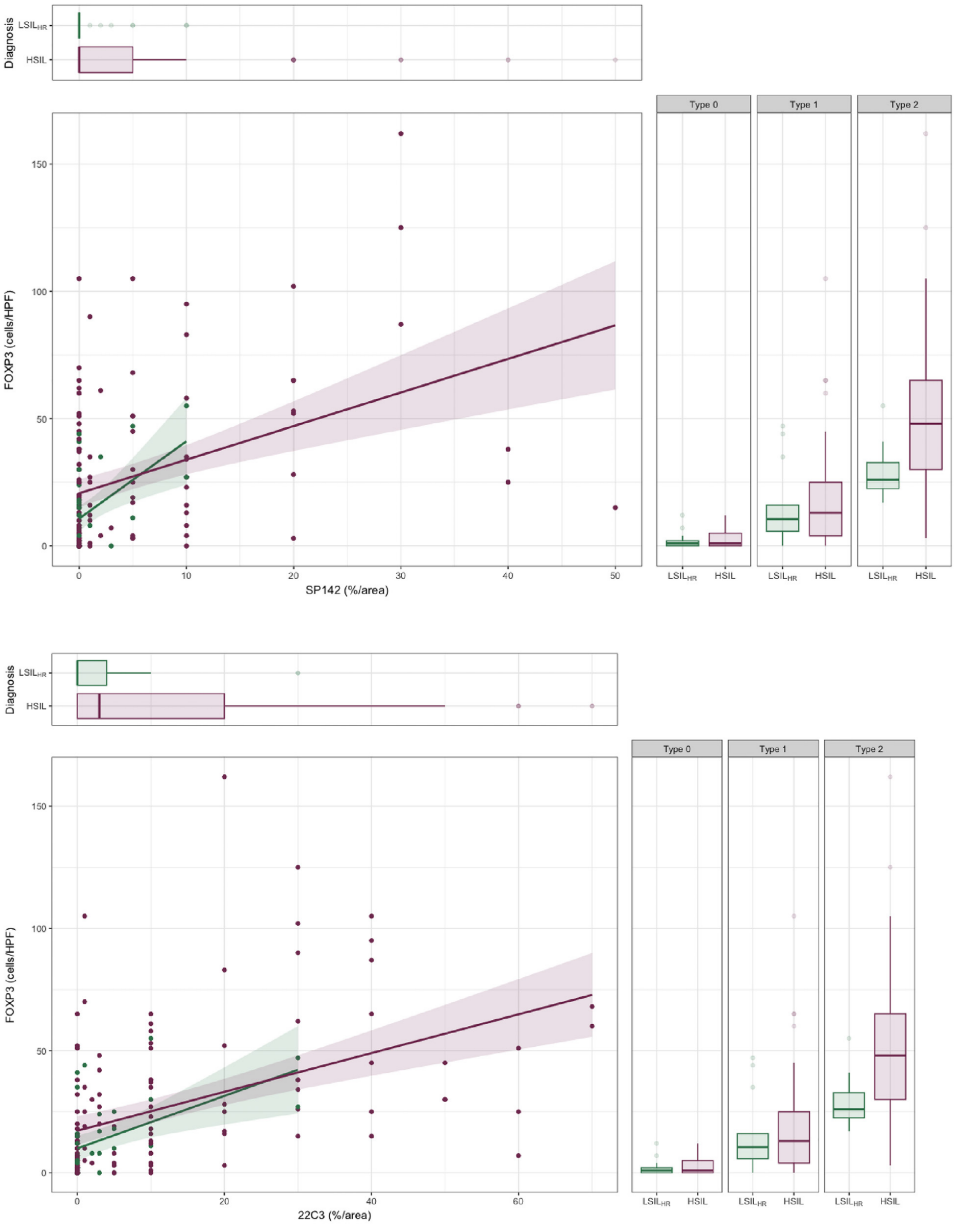
Comparison between HSILs (magenta) and LSILs_HR_ (green) according to inflammatory score, median count of FOXP3^+^ cells and PD-L1 score (SP142 top, 22C3 bottom). On the right, the boxplot shows differences in inflammatory scores and median count of FOXP3^+^ cells between HSILs and LSIL_HR_. Top left: median differences of PD-L1 expression between HSILs and LSILs_HR_. Bottom left: PD-L1 expression variation according to FOXP3^+^ cell counts. HSILs significantly presented higher inflammatory scores, FOXP3^+^ cell counts and PD-L1 expression than LSILs_HR_. Although a correlation between FOXP3^+^ cell counts and PD-L1 expression was observed in both groups, measurement differences could distinguish them.

PD-L1 expression was primarily detected in inflammatory cells (ICs); 9 (7.3%) HSILs exhibited focal staining in dysplastic epithelial cells (seven anal, one penile, and one vulvar lesion). While most LSIL_HR_ showed no stain or positivity in <5% of the infiltrate (*n* = 31, 88.5% and *n* = 26, 74.2% for PD-L1_SP142_ and PD-L1_22C3_, respectively), HSILs had a more significant proportion of lesions with staining in 5–49% or >50% of the IC (*n* = 37, 30.3% and *n* = 60, 49.1% for PD-L1_SP142_ and PD-L1_22C3_, respectively). A significant correlation was noted between PD-L1_SP142_ and PD-L1_22C3_ expression (r^2^ = 0.65, *p* < 0.05), and both clones were associated with HSIL diagnosis (*p* = 0.02 and < 0.01, respectively) and type 2 inflammatory score (*p* < 0.01 for both). Moreover, PD-L1_SP142_ and PD-L1_22C3_ expression correlated with the absolute FOXP3 count (r^2^ = 0.39 and r^2^ = 0.57, *p* < 0.01, respectively). The GLM showed that PD-L1_22C3_ expression was independently associated with HSIL diagnosis (*p*_adj_ < 0.01), higher inflammatory score (*p*_adj_ < 0.01), and FOXP3 count (*p*_adj_ = 0.03), while PD-L1_SP142_ was independently associated with HSIL diagnosis (*p*_adj_ < 0.01) and FOXP3 count (*p* < 0.01) (Figures 1–3).

### HIV status and immunodeficiency

HIV status was available for 31 patients (20.7%); 15 (10.0%) of them were HIV-negative (HIV-), and 16 (10.7%) were HIV-positive (HIV+), corresponding to a total of 34 cases. There were 29 anal lesions, including 21 HSILs (ten HIV+ and eight HIV- patients) and 8 LSILs_HR_ (five HIV+ and three HIV-), 3 vulvar HSILs (two HIV+ and one HIV-), and 2 penile HSILs (one HIV+ and one HIV-). Multifocal lesions (*n* = 14) were more frequent in the HIV+ (seven anal and two vulvar HSILs and 2 LSILs_HR_) than the HIV- group (two anal HSILs and one anal LSIL_HR_).

No significant associations other than multifocality (*p* = 0.01) were found between HIV+ status and other variables, such as age (*p* = 0.23), sex (*p* = 0.43), lesion size (*p* = 0.23), topography (*p* = 1.00), histological pattern (*p* = 0.90), ISH (*p* = 0.23), or p16 pattern (*p* = 0.69). In the study group, 3 patients had known immunodeficiency: one vulvar and one anal HSIL were diagnosed in patients with acquired immunodeficiency syndrome (AIDS), and one anal HSIL case occurred in a post-renal transplant setting. The main findings of patients with known HIV status are summarized in Table 4.

**Table 4:** Main findings of patients with known HIV status

	All patients	HIV status		*p*-value
		HIV+	HIV−	
Gender				
Male (%)	23 (74.2)	13 (41.9)	10 (32.3)	0.43
Female (%)	8 (25.8)	3 (9.7)	5 (16.1)	
Age (range)	32.0 (25.0 – 38.0)	33.0 (26.8 – 38.0)	26.0 (24.0 – 37.0)	0.23
	All cases			
Topography				
Anal	29 (85.3)	14 (44.1)	15 (41.2)	1.00
Penis	2 (5.9)	1 (2.9)	1 (3.0)	
Vulva	3 (8.8)	2 (5.9)	1 (2.9)	
Diagnosis				
HSIL (%)	26 (76.5)	14 (41.2)	12 (35.3)	0.68
LSIL_HR_ (%)	8 (23.5)	3 (8.8)	5 (14.7)	
Size (mm)	7.5 (3.0 – 12.8)	8.0 (5.0–13.0)	5.0 (2.0–13.0)	0.23
Focality				
Unifocal (%)	20 (58.8)	6 (17.6)	14 (41.2)	0.01
Multifocal (%)	14 (41.2)	11 (32.4)	3 (8.8)	
Infiltrate				
Type 0	8 (23.5)	5 (14.7)	3 (8.8)	0.58
Type 1	20 (58.8)	10 (29.4)	10 (29.4)	
Type 2	6 (17.7)	2 (5.9)	4 (11.8)	
FOXP3 (cells/HPF)	8.0 (3.0–25.0)	5.0 (1.0–15.0)	15.0 (7.0–30.0)	0.12
PD-L1 (22C3)^*^	0.0 (0.0–5.0)	0.0 (0.0–10.0)	0.0 (0.0–0.0)	0.11
PD-L1 (SP142)^*^	3.0 (0.0–10.0)	5.0 (0.0–20.0)	1.0 (0.0–10.0)	0.28

^*^PD-L1 expressed as continuous score.

No significant correlation was found between HIV status and the inflammatory score; type 1 inflammatory infiltrate was more often observed in HIV+ and HIV- cases (*n* = 10, 29.4% vs. *n* = 10, 29.4%, *p* = 0.58), followed by type 0 (*n* = 5) and type 2 (*n* = 2) in the HIV+ subgroup. However, all 3 patients with proven immunodeficiency had type 0 infiltrate. No significant difference was found in FOXP3 count between patients with HIV+ and HIV- status (5.0 (1.0–15.0) vs. 15.0 (7.0–30.0), respectively, *p* = 0.12). Similarly, comparison between PD-L1_22C3_ and PD-L1_SP142_ expression in patients with HIV+ and HIV- status showed no significant differences (0.0 (0.0–10.0) vs. 0.0 (0.0–0.0), *p* = 0.11 and 5.0 (0.0–20.0) vs. 1.0 (0.0–10.0), *p* = 0.28, respectively). Seven (41.2%) and 9 (52.9%) of the 14 cases of HIV+ HSILs showed PD-L1_SP142_ and PD-L1_22C3_ expression ≥5%, the latter including 6 patients with PD-L1_22C3_ positivity ranging from 20–70% of the infiltrate.

## DISCUSSION

This study demonstrated that the inflammatory response in a subset of anal, penile, and vulvar HSILs was associated with PD-L1 and FOXP3 expression. In addition, brisk inflammatory infiltrates and higher numbers of FOXP3^+^ and PD-L1^+^ cells were significantly more abundant in HSILs than in LSILs_HR_, suggesting the involvement of transcriptionally active HR-HPV in evoking inflammatory reactions and immune evasion at those sites.

In cervical HSILs, the intensity of the inflammatory reaction is associated with the risk of progression to SCC [33]. As the inflammatory response intensifies, the immune environment acquires a pro-tumorigenic nature by attracting tumor-associated macrophages, myeloid-derived suppressor cells, and FOXP3^+^T_regs_ [5, 33], thus overcoming the T_h1_ response. Concordantly, we found that, in non-cervical anogenital HSILs, the expression of immunosuppressive molecules was independently associated with dense infiltrates. One caveat in the interpretation of the inflammatory score in our series is that not all infiltrates were clearly physiopathologically related to the lesion and that few LSILs_HR_ had type 2 infiltrate (dense and continuous infiltrate filling at least two contiguous HPF = 40×). However, they developed into fistulae or were secondarily ulcerated, overestimating the score in those cases.

T-cell predominance occurred in all topographies; however, we found a proportion of anal lesions that showed more B cells due to the presence of tertiary lymphoid structures. Data on the number and size of these structures in normal individuals and their role in anal cancer are scarce [34]. Nevertheless, they were included as a part of the infiltrate as they were not oblivious to the presence of neoantigens in the microenvironment. Further comparisons between anal HSILs and HSILs from other sites could aid in determining if this peculiarity is associated with different biological behaviors.

In our series, HSILs had fewer CD8^+^ than CD4^+^ cells, considering intraepithelial and stromal T lymphocytes together. Studies on cervical SILs and SCC show predominance of CD4^+^ or CD8^+^ cells in the epithelium, stroma or in the lesion as whole [35, 36], and conflicting associations between the proportion of these T-cell subsets and regression, recurrence or progression have been reported. However, it is important to note that the characterization of different subpopulations within the CD8^+^ and CD4^+^ infiltrates seems to override the mere CD4:CD8 ratio assessment. For example, despite the known anti-tumor effect of CD8^+^ cytotoxic cells, a low CD4:CD8 ratio has been associated with worse 5-year survival rate in cervical SCC, likely due to the inactivated or underprimed status of CD8^+^ cells [37]. Similarly, CD4^+^ cells are not always T_h1_ cells which are necessary for the T-cell-mediated immune response against HPV; they are also represented by immunoregulatory T-cells (FOXP3^+^/CD25^+^) or PD1^+^ cells associated with immune evasion and lower rates of regression [38, 39]. In this study, the greater density of the infiltrate in the stroma, where CD8^+^ cells tend to be less numerous, and the high FOXP3 counts in our HSILs are likely responsible for the CD4>CD8 found.

Studies on FOXP3^+^T_regs_ in invasive neoplasms have yielded conflicting results, showing good or poor prognostic value for different tumor types or even for the same tumor type, as observed in penile and vulvar SCC_HPV+_ [20, 40–44]. Notably, some studies on FOXP3^+^T_regs_ in vulvar HSILs have shown that suppressing their activity may improve patient outcomes. One study reported a decrease in T_regs_ and an increase in the numbers of CD8^+^ effector cells and CD14^+^ myeloid cells after therapeutic vaccination in women with vulvar HSILs. Notably, this pattern was observed in the best responders, which were those with a previously well-established T_h1_ response [45]. In contrast, high number of T_regs_ cells and poor T_h1_ responses are observed in cases of ineffective local treatment of vulvar HSILs with immunomodulators such as imiquimod [46]. Although we found that HSILs had comparatively greater FOXP3+ numbers, we are yet to determine if these findings correlate with the outcome.

PD-L1 expression on tumor cells has been extensively explored in the literature. The fact that PD-L1 can also be expressed on T and B cells, macrophages, and dendritic cells has been less emphasized, even though it has an equivalent immune inhibitory function [23–25, 47–50]. In this study, PD-L1 staining was almost always observed in ICs, with few cases showing staining in epithelial cells. In one study wherein PD-L1 expression was assessed in anal intraepithelial lesions [29], 12% of the HSILs and 6% of the LSILs showed PD-L1 staining in epithelial cells, with no significant difference between the two groups. The same study verified that PD-L1^+^ lymphocytes had an equivalent distribution in high-grade and low-grade lesions. In contrast, our findings showed that the LSILs_HR_ with PD-L1^+^ cells had distinguishably lower scores, either categorical or continuous, barely comparable to HSILs.

PD-L1 expression in non-invasive neoplasms has been explored in parallel with its invasive counterparts, suggesting that immune evasion by PD-L1 starts early in dysplastic lesions and becomes more prominent with the achievement of the invasive phenotype [51]. In these examples, PD-L1 staining in epithelial and/or ICs gradually increased following tumor progression: from sessile serrated adenomas with low-grade dysplasia to colonic adenocarcinomas [28], from ductal carcinoma *in situ* to invasive ductal carcinoma of the breast [27], and finally from normal cervix/LSIL to HSIL, SCC, and metastatic SCC [31]. We observed a similar tendency when comparing PD-L1 expression in HSILs and LSILs_HR_. However, cases of SCC_HPV+_ were not included in the study.

A limitation of the present study is that information on the immunological status was available for only 3 patients. Therefore, a statistical comparison between immunocompetent and immunosuppressed individuals could not be established. However, it is interesting to note that all 3 patients with HSIL and known immunodeficiency had type 0 infiltrate. This is consistent with the fact that, theoretically, the immune inhibitory mechanisms induced by HR-HPV would only occur once there is a well-developed response by the adaptive immune system, which is impaired in immunocompromised patients. Notably, among patients with available HIV status, 64.2% of HIV+ HSILs cases had PD-L1 expression (22C3 and/or SP142 clones) >5% of the infiltrate, including 6 cases with PD-L1_22C3_ 20%. These findings indicate that, although patients with HIV+ status tend to present more frequently with multiple lesions, they can exhibit comparable immune responses to those with HIV- status, especially if they are receiving combined antiretroviral therapy (c-ARV) or do not manifest AIDS. One study, which included 166 patients with cervical SCC_HPV+_ and/or HSIL, assessed PD-L1 expression (SP263 and 22C3 clones) in women with HIV+ and HIV- status and found that 19% of HSILs non-adjacent to an SCC in patients with HIV+ status treated with c-ARV expressed PD-L1_SP263_ *versus* 0% of HIV-matched cases [51]. Further studies with a greater number of patients with HIV+/AIDS, and those with other types of immunosuppression would enable a more robust comparison.

Due to its descriptive nature, this study was unable to confirm the prognostic value of PD-L1 and FOXP3 expression in HSILs; whether they are clinically relevant biomarkers or solely represent an immune-exhausted phenotype in persistent HR-HPV infections should be assessed using a different methodology.

In conclusion, PD-L1 and FOXP3 are noticeably present in the immune environment of pre-malignant lesions of the anal canal, penis, and vulva induced by HR-HPV, similar to that described in cervical neoplasia and in other non-viral-associated pre-invasive neoplasms of other organs. Analyzing immune inhibitory biomarkers in this setting provides insights into their role in the early phases of carcinogenesis and creates the opportunity to investigate new approaches to identify distinct patterns of response and correctly approach high-risk patients, preventing overtreatment of lesions that may regress.

## MATERIALS AND METHODS

### Case selection

**Figure 4:**
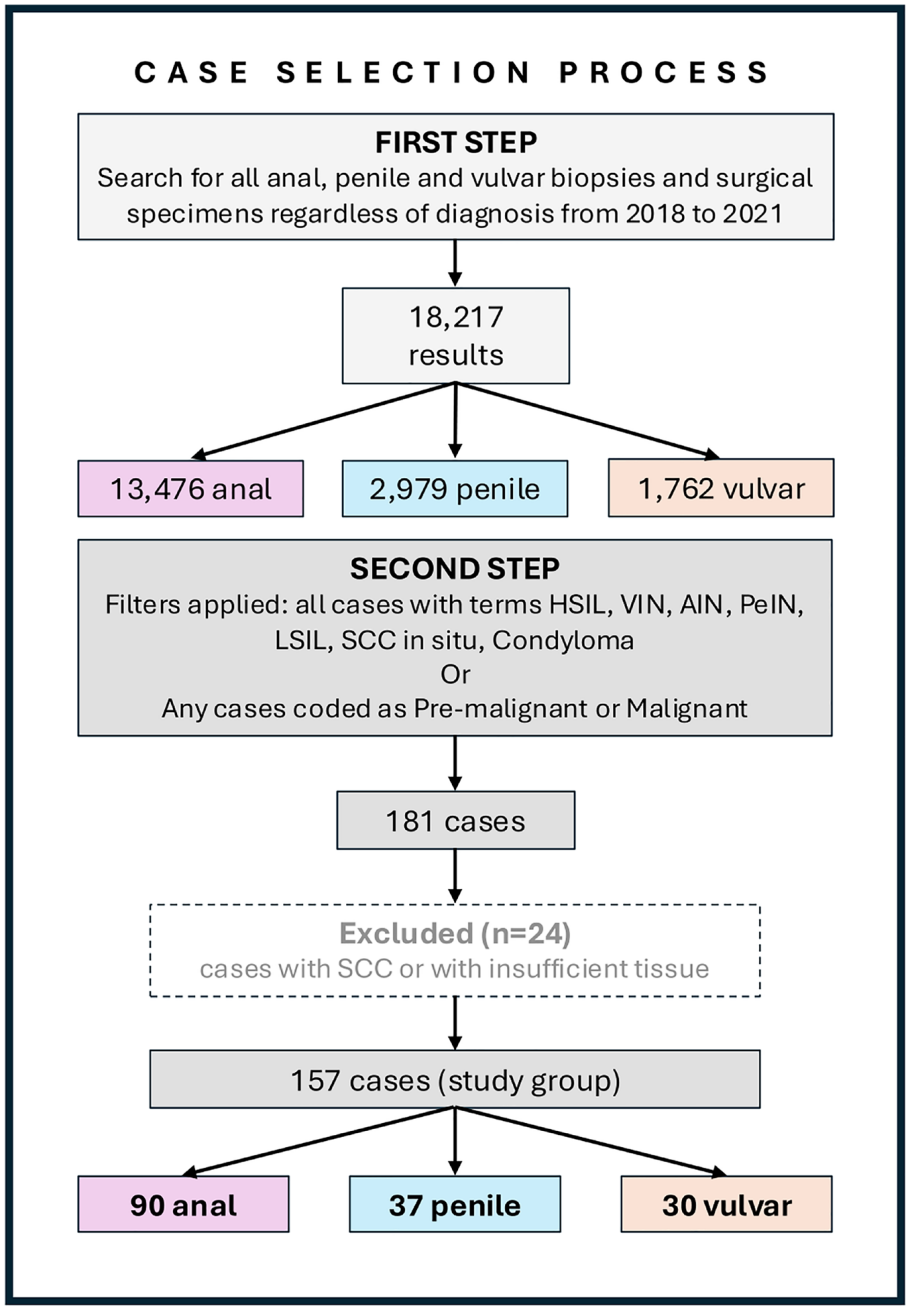
Case selection process diagram. Abbreviations: AIN: anal intraepithelial neoplasia; HSIL: high-grade squamous intraepithelial lesion; LSIL: low-grade squamous intraepithelial lesion; PeIN: penile intraepithelial neoplasia; SCC: squamous cell carcinoma; VIN: vulvar intraepithelial neoplasia.

An initial search using the topography of our database yielded 18,217 results (13,476 anal, 2,979 penile, and 1,762 vulvar samples), including biopsies and surgical specimens from 2018 to 2021. Then, a new search was conducted among these cases to find all pathology reports containing the terminology applied for intraepithelial lesions that are recommended by LAST [2], as well as “Squamous Cell Carcinoma *in situ*” and “Condyloma”. Furthermore, we searched for cases determined as “malignant” and “pre-malignant” in our coding system to ensure that cases classified with older terminology such as “Erythroplasia of Queyrat,” “Bowen`s Disease” or “High-Grade Dysplasia” were also included. Finally, we detected 181 cases of HSIL or LSIL co-infected with HR-HPV (LSIL_HR_), the latter including cases of LSIL in which ISH was performed at the time of the original diagnosis (Figure 4). Patients with concurrent SCC_HPV+_ or paraffin blocks with insufficient residual tissue for further sectioning were excluded from the study. When available, clinical data such as age, sex, size, focality, and recurrence status, as well as HIV infection and immune status, were retrieved from patients’ medical files.

### Histological assessment

Hematoxylin and eosin slides were examined, and once a consensus was achieved on the morphological diagnosis, we established the predominant architectural pattern of the lesion: flat, warty (synonymous with the bowenoid pattern for penile lesions), or condylomatous. Inflammatory infiltration in the dermis/lamina propria underneath or immediately adjacent to the lesion was regarded as a categorical variable:

- Type 0: No or few ICs with no aggregates or band-like foci.
- Type 1: aggregates or band-like pattern, discontinuous.
- Type 2: Dense and continuous infiltrate (that is, filling at least two contiguous HPF = 40×). Intraepithelial ICs were also counted.

### Immunohistochemistry

Immunohistochemistry was performed on Formalin-Fixed Paraffin-Embedded (FFPE) tissue using OMNIS and Autostainer Link 48 (DAKO) or BenchMark ULTRA (Ventana Medical Systems, Tucson, AZ, USA) automated platforms, following the manufacturer’s protocols for ready-to-use markers. A panel composed of CD3 (polyclonal, DAKO), CD4/CD8 double staining (SP35/SP57 rabbit monoclonal antibody, Ventana), CD20 (L26 mouse monoclonal antibody, DAKO), CINtec^®^ p16 (E6H4, Ventana), PD-L1 (SP142 Assay, Ventana), and PD-L1 (22C3, Agilent DAKO) was performed in one or more representative sections in each case. FOXP3 (EP340, rabbit monoclonal antibody, BioSB) was used at a 1:50 concentration with citrate-carbonate buffer for 64 min on a BenchMark ULTRA IHC/ISH platform.

P16 staining was interpreted as negative (no staining), block-type (“*continuous strong nuclear or nuclear and cytoplasmic staining of the basal cell layer with extension upward involving at least one-third of the epithelial thickness*” [2]), or focal (any staining that is not block-type).

Subsequently, CD3/CD20 and CD4/CD8 proportions were defined (CD3 >, or < CD20 and CD4 >, or < CD8), as well as the maximum number of FOXP3^+^ cells in one HPF (hotspot).

We assessed PD-L1 staining in mononuclear ICs, including lymphocytes, macrophages, and dendritic cells. Expression was calculated as the percentage of lesion area occupied by ICs with any discernible positivity. The score was categorized into four categories (negative, 1–4%, 5–49%, and 50%) but was also computed as a measurement variable.

### *In situ* hybridization

DNA ISH for high-risk HPV (HPV III Family 16 Probe, Ventana) was conducted on FFPE samples from all HSILs on the automated platform BenchMark ULTRA using the ISH iView Blue Plus detection kit (Ventana Medical Systems, Tucson, AZ, USA).

### Statistical analysis

Statistical analysis was performed using R Studio software [52]. The normality distribution of all continuous variables was assessed using the Shapiro–Wilk test. Categorical variables are expressed as frequency (%), while continuous variables are expressed as median and interquartile range (1st and 3rd interquartile range). Chi-square and Fisher’s exact tests were used to compare two categorical variables. Univariate analysis was performed using the Mann–Whitney and Kruskal–Wallis tests with Dunn’s post-hoc test and Bonferroni correction for comparison of continuous variables with categorical variables. Multivariate analysis was performed using a binomial or Poisson GLM. *P*-value < 0.05 was considered significant.

## Abbreviations

AIDS: acquired immunodeficiency syndrome
AIN: anal intraepithelial neoplasia
c-ARV: combined antiretroviral therapy
FFPE: formalin-fixed paraffin-embedded
FOXP3: forkhead box protein P3
FOXP3^+^T_regs_: FOXP3^+^ immunoregulatory T cells
GLM: generalized linear model
HIV: human immunodeficiency virus
HPF: high power field
HR-HPV: high-risk human papillomavirus
HSIL: high-grade squamous intraepithelial lesion
IC: inflammatory cells
ISH: *in situ* hybridization
LSIL_HR_: low-grade squamous intraepithelial lesions co-infected with high-risk HPV
NK: natural killer
PD1: programmed cell death Protein 1
PD-L1: programmed cell death-ligand 1
PeIN: penile intraepithelial neoplasia
SCC: squamous cell carcinoma
SCC_HPV+_: high-risk-HPV-associated squamous cell carcinoma
T_h1_: T helper 1 cell
VIN: vulvar intraepithelial neoplasia
